# Inflammation’s impact on the interaction between oligodendrocytes and axons

**DOI:** 10.1093/discim/kyaf008

**Published:** 2025-04-29

**Authors:** Tabitha R F Green, Marieke Pingen, Julia M Edgar

**Affiliations:** School of Infection and Immunity, College of Medical, Veterinary and Life Science, University of Glasgow, Glasgow, UK; School of Infection and Immunity, College of Medical, Veterinary and Life Science, University of Glasgow, Glasgow, UK; School of Infection and Immunity, College of Medical, Veterinary and Life Science, University of Glasgow, Glasgow, UK

**Keywords:** microglia, T cells, neurodegeneration, myelin, oligodendrocytes

## Abstract

Oligodendrocytes are responsible for the myelination of axons and providing trophic and metabolic support to the myelinated axon. They also interact with immune effector cells, including microglia and T cells, hence, are involved in central nervous system immune regulation. Given the crucial roles for oligodendrocytes and myelin in axonal function and maintenance, dysfunction, whether through cell death, myelin injury and loss, or failure in normal myelin formation, impairs neurological function. In diseases such as multiple sclerosis, the leukodystrophies, and viral infection, neuroinflammation is an important effector of myelin injury, having secondary consequences for the myelinated axon. In this review, we discuss the role of oligodendrocytes in health and inflammatory disease, with a focus on the interplay between inflammation and oligodendrocyte-axon interactions.

## Graphical Abstract

**Graphical Abstract:**
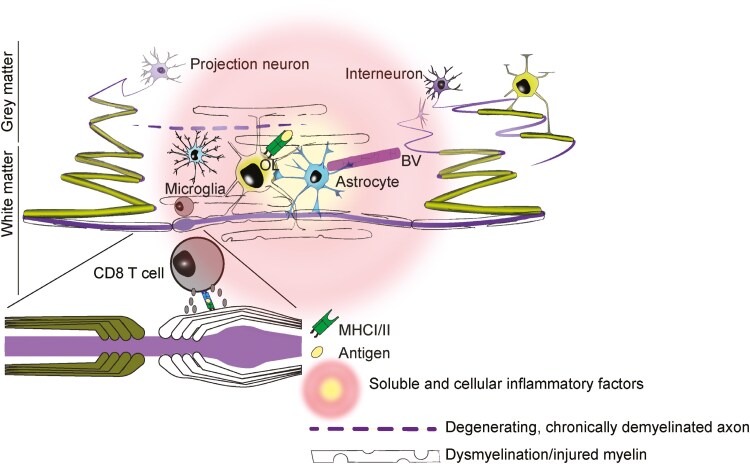

## Introduction

In the central nervous system (CNS), axons, the electrically excitable ‘cable-like’ processes of neurons, carry information from one nerve cell soma to another. Most CNS axons are surrounded segmentally by myelin, which is produced by oligodendrocytes. Myelin speeds up the propagation of neuronal signalling and likely helps establish and modify neuronal circuitry [1–4]. However, the role of oligodendrocytes extends beyond their crucial ability to myelinate axons, as described in detail below.

Oligodendrocyte cell death and subsequent myelin loss, myelin injury, or failure in normal myelination can each impair neurological function. Neuroinflammation is an important effector of oligodendroglial and myelin injury, both as a primary and secondary insult. In this review, we describe some diseases of CNS myelin and how the associated neuroinflammation affects the oligodendroglial-axonal unit. We also briefly describe the recent evidence that oligodendrocytes themselves can act as immune effector cells.

## Functions of oligodendrocytes

**Figure 1.**
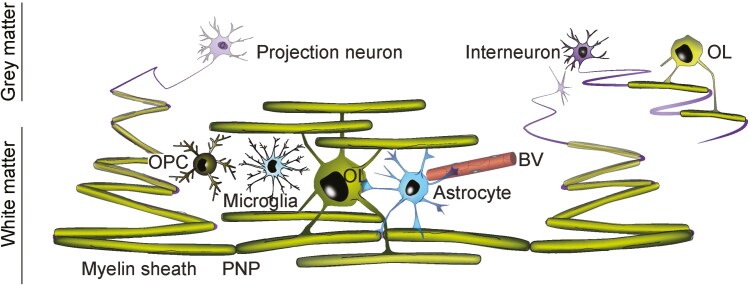
Grey and white matter myelin in the CNS. In the grey matter, some axons (purple, upper right) are intermittently myelinated (green). In white matter, most of the axon is sequestered beneath myelin, and myelin sheaths are separated only by short unmyelinated regions called nodes of Ranvier. In general, each oligodendrocyte (OL) myelinates multiple axons. The paranodes (P), where the paranodal loops of myelin attach to the axon through septate-like junctions, flank the node of Ranvier (N). Both grey and white matter harbour blood vessels (BV), astrocytes (blue), microglia (turquoise), and oligodendrocyte progenitor cells (OPCs). The last helps maintain immune homeostasis by downregulating microglial C-X3-C motif chemokine receptor 1 (CX3CR1) expression and suppressing microglial activation [5].

In the white matter of the CNS, myelin sheaths segment the axon into long (~20-200 μm) myelinated ‘internodes’ separated by short (~1 μm) unmyelinated nodes of Ranvier (Figure 1) where ion channels enable the ion flux required for action potential generation and transduction. In the grey matter, some axons are sparsely myelinated [6, 7] (Figure 1), and the function of this sparse myelin is less well understood and unlikely to be related to conduction velocity. Notwithstanding, a recent study showed that the sparse myelin on parvalbumin-positive γ-aminobutyric acid interneurons is crucial for effective inhibition of pyramidal neurons and enabling behavioural state-dependent modulation of local circuit synchronization in mice [2].

To understand how neuroinflammation impacts the oligodendroglial-axonal unit, it is important to highlight that the function of myelinating oligodendrocytes extends far beyond the insulation or modulation of neuronal circuits. Throughout life, oligodendrocytes deliver energy substrates and trophic factors to myelinated axons for axon energy homeostasis and survival (reviewed recently in Stassart et al. and Duncan et al. [8, 9]. At least in white matter tracts, this energy support function of oligodendrocytes overcomes the fact that myelin limits the capacity for such axons to receive energy substrates from the extracellular milieu, from which they are almost completely insulated [10, 11]. Myelinated axons require oligodendroglial-funnelled energy substrates, be they glucose, pyruvate, or lactate [12–14], for processes such as motor protein-dependent transport over long distances and to restore intracellular ion concentrations after action potential generation. The latter is particularly relevant as demonstrated by the relationship between ATP levels and compound action potential generation in an electrically stimulated *ex vivo* mouse optic nerve preparation [15].

Besides funnelling energy substrates, oligodendrocytes also buffer iron and extracellular potassium. For example, preventing the release of oligodendrocyte ferritin heavy chain-containing extracellular vesicles led to increased neuronal loss, through iron-mediated ferroptotic axonal damage, in mice [16]. Thus, oligodendrocytes have a crucial antioxidant defence function [16]. Conversely, Kir4.1 channels on the inner myelin membrane, facing the axon, sequester K^+^ released by firing axons, thus preventing seizure activity and maintaining axon health [17, 18]. Of note, activation of Kir4.1 channels by axon-derived K^+^ ions couples oligodendroglial metabolic support with axonal firing rate [19], a surrogate of axon energy consumption. Thus, periaxonal K^+^ ion concentration signals to the oligodendrocyte to match the axon’s fluctuating demands for energy substrates.

Oligodendrocytes also influence the structure of the myelinated axon. For example, they locally modulate neurofilament transport rates and phosphorylation, thus influencing local axon calibre, albeit to a lesser extent than in the peripheral nervous system [20–26]. Intriguingly, in grey matter, myelin clusters axonal mitochondria beneath the sparse myelin sheaths on interneuron axons [27]. However, in the optic nerve, a white matter tract that is continuously myelinated except at the optic nerve head, axonal mitochondria are most enriched at the unmyelinated region [27]. This latter pattern is seen also following white matter demyelination, where denuded axon segments have increased mitochondrial abundance compared to myelinated regions [28].

Together these data demonstrate the importance of oligodendrocytes for the function and health of myelinated CNS axons.

## Oligodendroglia as immune effectors and modulators

Neuroinflammation is an important effector of myelin and axon injury, both as a primary and secondary insult. Indeed, oligodendrocytes themselves can possess an ‘immune’ phenotype that could, in principle, actively contribute to disease pathogenesis, including axonal injury (reviewed in Castelo-Branco et al. [29]).

**Figure 2.**
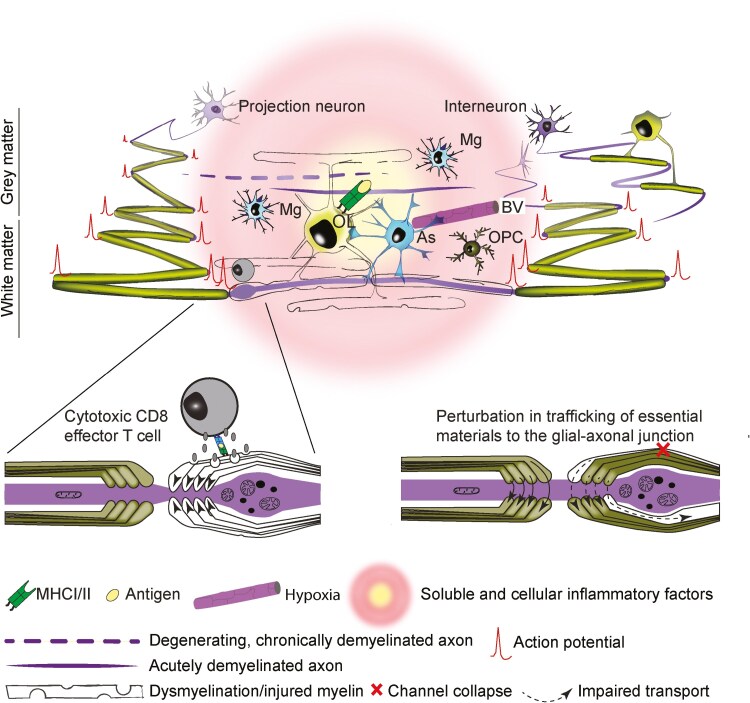
Oligodendrocyte dysfunction and axon injury in the inflamed CNS. Axons ensheathed by dysfunctional myelin are particularly susceptible to injury whether myelin dysfunction is due to gene mutation, as in the Plp1 and Cnp1 knockout mice, autoimmunity, as in MS and EAE, or viral infection, as in PML. While chronic demyelination can render axons vulnerable to degeneration, acutely demyelinated axons can survive for some time [35]. MHC-I and -II are induced in oligodendrocytes during inflammation and can mediate interaction with T cells. At the juxtaparanode, granzyme secreted by CD8 effector T cells is thought to induce actomyosin contractility at the paranodal loops (arrowheads, inset LHS), thus compressing the axon [36]. Organelle accumulation and axonal swelling, which resolves or leads eventually to axonal transection could alternatively be caused by reactive oxygen and nitrogen species acting on axonal mitochondria [37] or through collapse of the cytoplasm-filled spaces (pale green) of myelin [38] that mediate the transport of materials to the glial-axonal junction [39, 40]. OL: oligodendrocyte; Mg: activated microglial cell; As: astrocyte; BV: blood vessel; OPC, oligodendrocyte progenitor cell.

Oligodendrocytes express Toll-like receptors 2 and 3 which sense pathogen-associated molecular patterns and damage-associated molecular patterns such as dsRNAs, bacterial surface proteins, and high mobility group box1 protein [30, 31]. They also sense and secrete many immune-related chemokines and cytokines (reviewed in Kirby and Castelo-Branco [32]) and in some circumstances, express major histocompatibility complex (MHC)-I and/or -II [33, 34]. The major histocompatibility complex (MHC) is a large locus on vertebrate DNA that encodes various MHC proteins responsible for presenting antigen to T cells. MHC-I is displayed on all nucleated cells and presents mainly endogenous peptides, while MHC-II expression is largely restricted to thymic epithelial cells and antigen-presenting cells and presents mainly exogenous antigens. Intriguingly, bulk and single-cell RNA-sequencing of neural cells demonstrated induced expression of MHC-I and -II genes in mouse oligodendroglia (including myelinating oligodendrocytes; Figure 2) in experimental autoimmune encephalomyelitis (EAE) and/or cuprizone-induced demyelination [41]. As in various stromal cells [42], interferon-γ is sufficient to induce MHC-II protein expression in oligodendrocytes and their progenitors (OPCs) and oligodendrocytes *in vitro* [34]. Thus, antigen presentation by oligodendroglia in neuroinflammatory conditions represents a potential direct route of interaction between oligodendrocytes and T cells (Figure 2), having implications for myelin degeneration and axonal injury [34, 43, 44].

As well as acting as immune effector cells themselves, OPCs modulate the principal immune effector cells of the CNS, microglia [45], the long-term activation of which can incite a chronic pro-inflammatory microenvironment (reviewed recently in Green and Rowe [46]. For example, in the healthy brain, OPCs help maintain immune homeostasis through the transforming growth factor (TGF)-β2-TGFβR2 axis, which downregulates microglial C-X3-C motif chemokine receptor 1 CX3CR1 expression and suppresses microglial activation [5]. Recent publications highlight the roles microglia can play in myelin development, damage, dysregulation, and repair, thereby directly influencing neuron function and survival [47–49]. Together, the immune phenotype of oligodendroglia and the bidirectional interaction with microglia highlights their role as important cellular players in neuroinflammation.

## Myelin disease and neuroinflammation

As indicated in the section ‘Oligodendrocyte Function’, oligodendrocytes and myelin play a crucial role in axonal function and maintenance. In the following sections, we briefly describe some diseases of CNS myelin and how the associated neuroinflammation affects the oligodendroglial-axonal unit. Ageing is not discussed here, having been reviewed recently [50], but there is considerable evidence that inflammation contributes to injury of myelinated axons in the aged CNS [51, 52] and that age-dependent oligodendrocyte dysfunction contributes to the pathogenesis of Alzheimer’s disease [53].

## The leukodystrophies

The leukodystrophies are a group of diseases that primarily affect white matter. They are caused by gene mutations leading to dys- and/or demyelination accompanied by secondary neuroinflammation. Pelizaeus Merzbacher disease (PMD) is a well-characterized hypomyelinating leukodystrophy, caused by a mutation in the proteolipid protein (*PLP*) gene. Although PMD is generally considered non-inflammatory, dys-and demyelinating animal models of PMD caused by gene overexpression (modelling gene duplication in patients, the most common cause of PMD) have overt microglial activation [54, 55] and small numbers of pathogenic CD8 + effector T cells in the CNS [56–58]. Crucially, these T cells are closely associated with MHC-I expressing ‘mutant’ oligodendrocytes, as revealed by confocal microscopy [57]. When in close proximity to a target cell, CD8 + T cells can excrete perforin which forms pores in the target cell, resulting in the uptake of granzymes, which induces apoptosis. Indeed, in *Plp1* overexpressing mice, neural damage could be partially ameliorated by chimerizaton with bone marrow from mice deficient in perforin or granzyme B, illustrating the importance of this mechanism for neuronal injury secondary to primary genetic abnormalities in oligodendrocytes [59]. The role of CD8 + effector T cells in axonal injury is described further in the section **‘**How neuroinflammation disrupts glial-axonal interaction’.

In contrast to the *Plp1* overexpressing mouse, the *rumpshaker* mouse (point mutation in the *Plp1* gene; Ile^186^Thr), a model of the allelic disorder spastic paraplegia type 2, has only a mild neuroinflammatory response characterized by a slight increase in microglial abundance in white matter tracts where myelin is thinner than normal, but otherwise grossly normal in appearance. Here, Wallerian degeneration affects only the longest myelinated axons in aged mice, indicating a slowly progressing, length-dependent mechanism that could be causally related to mild neuroinflammation, failure of oligodendroglial-mediated axonal support, or both [55, 60]. Remarkably, despite the pathogenic immune response in *Plp1*-overexpressing mice, a ketogenic diet can ameliorate clinical outcomes and axon and myelin pathology, associated with reduced endoplasmic reticulum stress and restoration of axonal mitochondrial morphology [61]. In the same model, wild-type neural stem cells can outcompete the endogenous ‘mutant’ cells to restore normal myelin following transplantation into the juvenile brain [58]. Notably, the ketogenic diet did not reduce microgliosis or astrocytosis, suggesting it did not reduce neuroinflammation [61], whereas neural stem cell transplantation was associated with a local reduction in activated microglia/macrophages [58].

Distinct from PMD, patients with genetic metabolic leukodystrophies such as X-linked adrenoleukodystrophy (X-ALD) or metachromatic leukodystrophy (MLD), due to mutation in the ATP binding cassette subfamily D member 1 gene and the arylsulfatase A gene respectively, may present at any age with extensive gadolinium-enhancing tumefactive lesions, associated with inflammatory demyelination, neuronal loss, and blood-brain barrier leakage [62]. Significantly, although oligodendrocytes are generally considered key to the pathophysiology of X-ALD and MLD, recent data convincingly demonstrate that microglial injury and loss precedes oligodendrocyte decay and overt myelin loss in these diseases [63]. Thus, the extent of neuroinflammation associated with the leukodystrophies varies across the disorders and within allelic disorders, with overt demyelination being associated with more severe neuroinflammation than hypomyelination, and also with a greater degree of axon injury.

Intriguingly, genetic alterations that primarily affect microglial also result in white matter abnormalities in adult-onset leukoencephalopathy with axonal spheroids and pigmented glia (ALSP), a rare human neurodegenerative disorder [64]. ALSP is due to mutations in the colony-stimulating factor 1 receptor (*CSF1R)* gene, which encodes CSF1R, a receptor for CSF1 and interleukin 34, required for the survival of microglia. ALSP was recently modelled by specifically ablating microglia in mice through knockout of the conserved *fms* intronic regulatory element, *Fire*, from the *Csf1r* locus, leading to age-dependent focal demyelination through dysregulation of TGFβ1-TGFβR1 signalling [48].

## Viral infection

Viral infection is one of the leading causes of demyelination in humans and animals [65], either due to the inflammatory response to infection or through direct infection of oligodendrocytes The latter is exemplified by progressive multifocal leukoencephalopathy (PML), an often-fatal demyelinating disease of humans [66] caused by human polyomavirus 2 (HPyV-2, previously known as John Cunningham or JC virus) which infects oligodendrocytes and astrocytes following the release of virus-carrying extracellular vesicles from the choroid plexus [67]. PML has become increasingly common in the last 40 years in parallel with the acquired immunodeficiency syndrome epidemic and the use of immunosuppressants for autoimmune diseases such as rheumatoid arthritis and Crohn’s disease. One such immunosuppressant is natulizumab [68], a monoclonal antibody that targets α4-integrins which are required for cell migration, for the treatment of MS and other autoimmune disorders. Approximately 80-90% of the human population is seropositive for HPyV-2 and it remains unclear whether immunosuppression allows virus replication in the periphery with subsequent spread to the brain or whether HPyV-2 is present in the brain and immune responses are overwhelmed upon immunosuppression. Demyelination in PML is accepted to result from lytic viral infection of oligodendrocytes (reviewed in Ono et al. [69]). Notably, in the preclinical and early phases of PML, HPyV-2 causes extensive neuronal damage, although it remains unknown whether this is due directly to glial cell infection, or indirectly through the immune reconstitution inflammatory syndrome [70].

Direct viral infection of oligodendrocytes is also observed in animal models, including laboratory mice. For example, Theiler’s murine encephalomyelitis virus (TMEV), Semliki Forest virus, and Zika virus infect oligodendrocytes and other neural cells in mice [71–75]. Not only can these viral-mediated demyelinating models help us understand the pathogenesis of human disease, so too can they help us understand oligodendroglial-axonal interaction. For example, Theiler’s virus has taught us that axonal content can be transferred to the glial cell, probably at the paranodal glial axonal junction where the two cells abut each other [73, 76, 77]. Theiler’s virus represents a remarkable example of a pathogen that navigates the various cells of the organism to evade immune responses and establish a persistent infection. Infection of oligodendrocytes is crucial for Theiler’s virus to persist in the CNS, which it can achieve by transferring non-lytically from the axon [73, 77].

Demyelination following infection with neurotropic virus can alternatively be caused by aberrant immune response to the infection. For example, in some mouse models, T and B cells play a critical role in demyelination after viral infection, for instance with a neurovirulent strain of Murine Hepatitis Virus [78], TMEV [79, 80], or Semliki Forest Virus [80–82]. Intriguingly, even in the absence of detectible virus in the brain, white matter-selective reactive microglia and increased levels of cytokines/chemokines in cerebrospinal fluid accompanied a loss of oligodendrocytes and myelin in a mouse model of SARS-CoV-2 infection, possibly helping explain cognitive impairment in some survivors of COVID-19 in whom white matter-selective reactive microglia were also observed [40]. The complex balance between protective and harmful adaptive antiviral immune responses in the CNS remains incompletely understood.

## Multiple sclerosis

Acquired CNS neuroinflammatory disorders are caused by immune dysfunction. Such diseases include multiple sclerosis (MS), acute disseminated encephalomyelitis, neuromyelitis optica spectrum disorder, and myelin oligodendrocyte glycoprotein-associated disorders. MS is the best-known inflammatory demyelinating disease of the CNS and the leading cause of neurological disability in young adults across many countries in the world. In most cases, MS starts as a relapsing–remitting disease that gradually transitions into a progressive phase, where symptoms gradually worsen without distinct relapses and remissions. Several recent reviews describe the pathological events thought to underlie MS [83–85].

Although the aetiology remains unconfirmed, MS is generally considered autoimmune in nature (the outside-in hypothesis), with immune cells activated in the periphery crossing the blood-brain barrier and attacking CNS myelin [86]. One putative external factor is viral infection during adolescence. Several lines of evidence suggest that infection with Epstein–Barr virus [87] triggers an autoimmune response against glial proteins through molecular mimicry [88, 89]. The alternative possibility that MS begins with a primary pathogenic event in the CNS (the inside-out hypothesis), that could, in susceptible individuals, lead to secondary pathogenic inflammation [90], remains a matter of topical debate [91].

Histopathologically, MS is characterized by extensive grey matter demyelination, especially close to the pia [92] and focally demyelinated ‘plaques’ in the white matter, as well as histological and imaging features consistent with remyelination [93–95]. T and B lymphocytes and myeloid cells infiltrated from the periphery are associated with these lesions. For more than a century, the histological features of grey and white matter lesions have been described, culminating recently in an updated histological classification system [96] which highlights the molecular and cellular changes observed at various stages of lesion development and disease progression. Aside from various pathological changes in myelin and oligodendroglia, axon injury and loss are a key feature of MS and, alongside the loss of neuronal cell bodies, the principal correlate of permanent neurological disability in progressive MS.

Restoration of myelin to demyelinated axons is widely considered crucial to protecting neurons in MS [97]. Given the topic of this review, it is worth noting that microglia have been shown to enhance (re)myelination through a variety of mechanisms including clearing myelin debris [98] secretion of Activin A [99, 100] supply of lipid and cholesterol to oligodendrocytes [101] and through fractalkine (CX3CL1; C-X3-C motif ligand 1) signalling [102]. The role of microglia in remyelination and in MS more generally is complex, and the subject matter has been reviewed recently [103, 104].

## How neuroinflammation disrupts glial-axonal interaction

In the sections above, we highlighted some disease-specific examples in which primary or secondary neuroinflammation causes injury to axons through its actions on the oligodendrocyte and myelin. Publication of key manuscripts late last century reinvigorated efforts to understand how axons are injured in myelin disease including MS and the leukodystrophies [105–113]. In MS, axon injury has been attributed, singly or in combination, to soluble and cellular immune-mediated inflammatory insult, altered sodium channel function, axonal energy insufficiency, or loss of trophic/metabolic support from the oligodendrocyte. Although most current research focusses on the susceptibility of demyelinated axons in MS, data from leukodystrophy mouse models that develop axon injury while retaining (almost) normal levels of myelin [111–113] show that myelin per se does not preserve axons. These observations, in myelin gene knockout mice, highlight that molecularly impaired myelin presents a risk for axonal health. Could acute demyelination preserve axons in circumstances where oligodendroglia or myelin are impaired, for example, due to cellular or soluble inflammatory factors?

We previously showed that, in the same optic nerve, axons undergoing active, genetically induced demyelination were more susceptible to injury (focal swelling and axonal transport stasis) than regions of the same axons that were already completely demyelinated [55]. More recently, a study led by the Stassart and Nave laboratories showed that in MS, EAE, and cuprizone-induced demyelination, both demyelinated and/or non-myelinated spinal cord axons were protected from irreversible axon damage compared to their myelinated neighbouring axons, even when the two populations shared the same inflammatory milieu [114]. Simultaneously, a manuscript from the Martini laboratory showed that the efficient stripping of dysfunctional myelin (point mutation in the *Plp1* gene) protected axons against T cell-driven degeneration, mechanistically related to aberrant actomyosin constriction of axons at paranodal regions [36] (Figure 2). In the last of these three studies, the importance of adaptive immune cells in axonal demise was demonstrated by crossing the *Plp1* mutant mice to *Rag1* knockout mice, which lack functional lymphocytes. In the double mutants, axonal loss was significantly attenuated, although not completely ameliorated [36]. Together these data demonstrate the importance of inflammation in axonal injury in the context of inflammation-induced perturbation of oligodendrocytes and myelin, whilst also confirming the importance of molecularly intact oligodendrocytes for axonal survival.

Intriguingly, in adult-onset cerebral ALD, extensive and severe acute axonal damage can be observed prior to overt demyelination in prelesional areas defined by microglia loss and relative myelin preservation [115].

These recent data expand upon previous observations demonstrating that in EAE, an animal model of inflammatory demyelination, the earliest stages of ‘focal axonal degeneration’ (FAD) are observed on axons with intact myelin sheaths [37]. FAD begins with focal swelling of the myelinated axon (Figure 2), which can either resolve or progress to fragmentation of the axon. Mechanistically, Nikić et al. [37] demonstrated that in EAE, macrophage-derived reactive oxygen and nitrogen species (ROS and RNS) could trigger pathological changes in axonal mitochondria and initiate FAD. Indeed, they found that neutralizing ROS and RNS rescued axons already entering the degenerative process. These data are compatible with an earlier report demonstrating that the application of nitric oxide to electrically active myelinated fibres led to axon degeneration in rats, highlighting that action potential generation itself contributes to the axon’s vulnerability to inflammatory factors [116], possibly by depleting energy substrates.

Together, these studies demonstrate how myelinated axons are impacted by cellular and soluble inflammatory factors that primarily target myelin, either following immunization with myelin peptide, as in EAE, or due to mutation in genes expressed in oligodendrocytes, as in the leukodystrophy models.

## Conclusion

Oligodendrocytes are complex cells that have many essential homeostatic functions: myelination, axonal trophic support, and ion buffering among others. Under pathological conditions, oligodendrocytes can sense and emit cytokines and chemokines and express MHC molecules, making them key players in neuroinflammatory disease of the CNS. Direct interactions between oligodendrocytes and microglia, T cells, and/or their products, have been shown to impact directly on axonal health. Thus, besides myelin dysfunction (due to mutation in genes expressed by oligodendrocytes or chronic demyelination), inflammation-induced myelin injury itself poses a risk for axonal health in diseases like MS, the leukodystrophies, and viral infection. The findings presented in this review highlight the need for more research to understand how oligodendrocyte functions in the context of axonal support are impaired by inflammation. One putative mechanism not already mentioned in this review is through disruption of transport processes within the cytosolic spaces of myelin that are likely vital to the transfer of materials between the two cells [39, 117] (Figure 2).

## Data Availability

Not applicable.
