# Correction to: Inflammation's impact on the interaction between oligodendrocytes and axons

**DOI:** 10.1093/discim/kyag005

**Published:** 2026-03-07

This is a correction to: Tabitha R F Green, Marieke Pingen, Julia M Edgar, Inflammation's impact on the interaction between oligodendrocytes and axons, *Discovery Immunology*, Volume 4, Issue 1, 2025, kyaf008, https://doi.org/10.1093/discim/kyaf008

The originally published version of this manuscript has been amended to correct the following errors.

In the “Author contributions” section, author Julia M. Edgar's contributions have been corrected to include the role “Visualization”.

Additionally, in the following sentence in the section entitled “Functions of oligodendrocytes”, the unit “mm” should have been given as “µm”:

“In the white matter of the CNS, myelin sheaths segment the axon into long (∼20-200 mm) myelinated ‘internodes’ separated by short (∼1 mm) unmyelinated nodes of Ranvier (Figure 1) where ion channels enable the ion flux required for action potential generation and transduction.”

This has been corrected and the updated sentence reads:

“In the white matter of the CNS, myelin sheaths segment the axon into long (∼20-200 µm) myelinated ‘internodes’ separated by short (∼1 µm) unmyelinated nodes of Ranvier (Figure 1) where ion channels enable the ion flux required for action potential generation and transduction.”

